# Effect of Thermal Ageing on Flexural Strength and Microhardness of Novel High-Performance Polymer (Nanoksa G-Plus) in Comparison to a Widely Used Bio-HPP/PEEK

**DOI:** 10.3390/dj13080370

**Published:** 2025-08-15

**Authors:** Ramy Abdallah Abdelrahim, Ahmed Ali Ezzeldine, Mahmoud Abdellah, SaadEldein Sadeq Elghazawi

**Affiliations:** 1 Faculty of Dentistry, Al-Azhar University, Cairo 11651, Egypt; ahmedramadan209@azhar.edu.eg (A.A.E.); mahmoudrefaei.46@azhar.edu.eg (M.A.); drsaadeghzawy@iunajaf.edu.iq (S.S.E.); 2 Dental College of Dentistry, The Islamic University, Najaf 54001, Iraq

**Keywords:** flexural strength, microhardness, Nanoksa G-Plus, PEEK, thermal ageing

## Abstract

**Background/Objectives:** The dental industry is continuously developing high-performance polymer (HPP) materials with different qualities for denture frameworks. The aim of this in vitro study was to assess how thermal ageing (TA) affects the flexural strength (FS) and microhardness of two different HPP materials: Nanoksa G-plus and Bio-HPP/PEEK. **Methods:** The TA process was carried out for 5000 cycles at 5 °C and 55 °C in distilled water. To assess FS, a total of 40 bar-shaped specimens measuring 65.0 mm × 10.0 mm × 2.5 mm (20 per group) were obtained; TA and No-TA (NTA) subgroups were prepared for each material group (10 per subgroup); and a three-point bending test was conducted using an Instron universal testing machine. Each specimen that fractured during the FS test was subjected to microhardness measurement using a Vickers hardness tester. The mean FS and microhardness of the TA and NTA specimens were statistically examined using the *t*-test. **Results:** Both the TA and NTA Bio-HPP/PEEK specimens exhibited significantly greater (*p* < 0.0001) microhardness and FS qualities than the Nanoksa G-Plus specimens. The FS and microhardness of the Bio-HPP/PEEK and Nanoksa G-Plus materials significantly decreased (*p* < 0.05) after TA. **Conclusions:** The Bio-HPP/PEEK material showed better FS and microhardness properties than the Nanoksa G-Plus material. TA considerably decreased the FS and microhardness of the Bio-HPP/PEEK and Nanoksa G-Plus materials.

## 1. Introduction

Progress in removable partial denture innovation is closely linked to the advancement of dental materials. Metal alloys are often used in removable partial denture frameworks due to their precision, strength, retention, and friction. However, rust is a significant drawback [1]. The use of poly-ether-ether-ketone (PEEK) as a metal-free alternative HPP material provides new possibilities for prosthetic applications [1,2,3].

PEEK is a substantial polymeric material in the field of prosthetic dentistry. It is a semicrystalline linear polycyclic aromatic thermoplastic polymer that demonstrates unique physical and chemical stability characteristics [2,4,5]. In its pure state, PEEK is mechanically weak, and PEEK clasps are not as strong as metal alloy clasps [4,6].

Industrially fabricated pre-polymerised blocks and discs with a uniform structure are used in CAD/CAM technology to improve the mechanical strength and long-term stability of restorations. Moreover, in processed CAD/CAM materials, the internal and exterior flaws produced when using laboratory processes such as polymerisation are reduced compared to those produced when using traditional procedures [7].

Composite resins reinforced with nanoceramics are hybrid dental materials that combine the optical and physical qualities of dental ceramics and polymer materials. In terms of microstructure, they have an organic polymeric matrix that is strengthened by ceramic inorganic crystals such as zirconium dioxide and quartz. The material’s mechanical properties are enhanced by ceramic crystals, increasing its resistance to wear and breakage [8].

Nanoparticle-filled polymeric nanocomposites have unique properties due to their large surface area, which increases the interaction between the matrix and reinforcement. The properties of nanocomposites make them suitable for high-performance applications. Previous studies have found that metallic or inorganic nanoparticles may be efficiently employed as a reinforcement for both categories of polymeric matrix materials: thermoplastic and thermosetting polymers [9,10,11].

The mechanical characteristics of PEEK can be improved by developing modified materials with fillers [4,6]. PEEK with 20% ceramic fillers, termed Bio-HPP-PEEK, is highly biocompatible and non-allergic. The capacity to make adjustments, superior stability, and maximum polishability of Bio-HPPs contribute to the creation of superior prosthetic restorations [1,2,12].

High-performance PEEK is a member of the polyaryletherketone (PAEK) family of polymers. Thermo-pressing techniques are used to create Bio-HPP devices. HPP devices can also be milled using computer-aided design/computer-aided manufacturing (CAD/CAM) techniques. Because of their low translucency and opaque coloration, which can be characterised as either greyish or pearl-white, HPPs are inappropriate for use in monolithic aesthetic dental repairs. Therefore, a veneer made of a resin composite might be necessary to achieve a satisfactory degree of aesthetic appeal [13].

Nanoksa G-Plus is a blend of biocompatible high-performance polymers (mainly methacrylates) containing nano-carbon and nano-zirconia, and it is used to create long-lasting restorations on dental implants. Nanoksa G-Plus was introduced by the manufacturer as a complete system that provides final solutions for restoring dental implants; whether it be a crown, bridge, or full-arch denture, the significance of this hybrid carbon is emphasised [14,15].

Nanoksa G-Plus is presented by the manufacturer as a resin or disc: the resin is indicated for use in veneers, inlays, onlays, and single crowns [14], and the disc absorbs very little water—roughly 0.1% after immersion in water for 24 h at 23 °C according to the manufacturer. Because of its very crystalline structure, Nanoksa G-Plus is insoluble in ordinary solvents at ambient temperature. According to the International Organization for Standardization (ISO) 527:2019 [16] and ISO 178:2019 [17], its yield strength is 90–100 MPa, and its breaking load in tensile tests is approximately 95–110 MPa [18]. Additionally, the disc is made to work with a range of materials, such as composites, ceramics, and metals. The primary advantage of this material is its special composition, which com-bines stress absorption and flexibility [18].

Nanoksa G-Plus is composed of a unique combination of polymers that are in-tended to withstand wear and breakage while offering a consistent smooth cut. Ac-cording to the manufacturer, Nanoksa G-plus is distinguished by its remarkable strength characteristics, outstanding biocompatibility, monomer-free nature, low weight, mechanical stability, long-term durability, and micro-mechanical elasticity (shock absorption). It is a material with high aesthetics and transparency that mimics the colour of natural teeth, and it has excellent abrasion resistance and colour stability. The goal was to create a material with a high strength and a high flexural strength comparable to those of PEEK [14].

Zirconia is used in dental restorative materials for its tooth-like colour and enhanced strength [19]. Adding zirconia nanoparticles to the resin matrix of polymers increases mechanical performance due to excellent interfacial bonding and substantial filler strength [9]. Furthermore, it has been reported that adding carbon fibre reinforcement to high-performance polymers enhances their strength [20].

The long-term viability of a repair depends critically on its flexural strength. The endurance of CAD/CAM restorations, as well as the behaviour of the restoration in response to flexion under occlusal stress, may be predicted through in vitro flexural strength testing [7]. The ability of a material to withstand repeated indentation or penetration is known as its hardness [21]. Hardness is employed to demonstrate a material’s resistance to abrasion and wear [22]. Restorations are impacted by daily wear, both during cleaning and usage. Given that wear is caused by abrasion, surface hardness is crucial [23]. Because of its connection to other physical attributes, hardness is a mechanical property that should be taken into account when examining restorative materials [24].

One reliable technique for simulating the ageing of restorative materials in vitro is thermocycling. The intraoral temperature fluctuations caused by consuming hot and cold foods and beverages are represented by the thermal changes that occur when performing a thermocycling test. The cracks and mechanical tensions caused by these temperature changes may indicate how long the restorations will last [25].

Dental restorations may experience thermally induced volumetric strains as a result of intraoral thermal challenges, particularly when composite and polymeric mate-rials with different thermal expansion coefficients are used [7]. There is little information available on the flexural strength, hardness, and durability of new CAD/CAM HPP materials. As far as the authors are aware, no previous studies have examined the flexural and hardness properties of Nanoksa G-plus HPP materials meant for the construction of denture frameworks or assessed how they respond to thermal ageing while using the same specimen size.

It is essential to analyse all pertinent possibilities in order to guarantee that the restoration materials used are appropriate for the work [7,26]. This entails investigating the durability and mechanical properties of various materials, as well as considering the risks and intended usage of each choice. By following these procedures, problems or complexities that might result from using the wrong materials for CAD/CAM-manufactured frameworks can be avoided [7].

Therefore, the aim of this study was to compare the recently developed high-performance polymer Nanoksa G-plus to the commonly used high-performance polymer Bio-HPP/PEEK in terms of hardness and flexural strength both before and after thermal cycling.

The first null hypothesis stated that the recently developed HPP Nanoksa G-plus and the widely used HPP Bio-HPP/PEEK had the same hardness and flexural strength before and after thermal ageing, and the second null hypothesis stated that thermal ageing would not change the properties of the different high-performance polymeric materials.

## 2. Materials and Methods

### 2.1. Study Design

**Figure 1:**
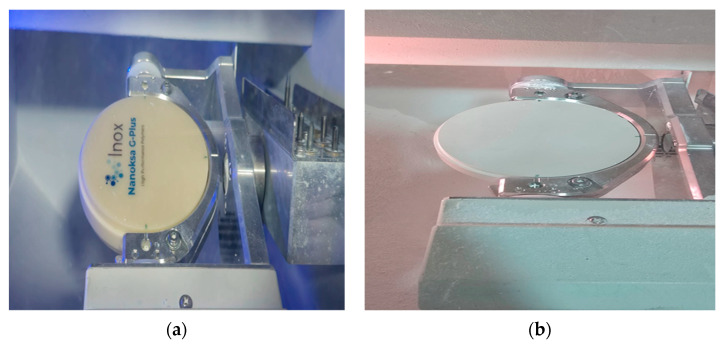
The two high-performance polymer materials utilised in this study: (**a**) Nanoksa G-Plus CAD/CAM block (**b**) and Bio-HPP/PEEK CAD/CAM block.

Two CAD/CAM blocks made from HPP materials for framework fabrication were investigated in the present study: Nanoksa G-plus (Nanoksa G-plus, INOX MENA Co., U.S. Dental group, 14979 Prairie Ave, Lawndale, CA, USA) and Bio-HPP/PEEK (partially crystalline PEEK with ceramic fillers, Bredent group GmbH & Co. KG, Senden, Germany) (Figure 1).

The sample size in similar previous research was examined, which revealed notable variations in comparable sample sizes [7,27]. Using the findings of prior research and the online statistical programme G*Power version (3.1.9.7) for Windows, a sample size of 10 per test was determined to have 80% power (alpha = 0.05) for detecting significant variations in flexural strength [28].

For the FS test, a total of 40 bar-shaped specimens (n = 20) were obtained; addition-ally, each specimen that fractured during the FS test was subjected to microhardness measurement. Thermal ageing (TA) and No-TA (NTA) subgroups were created for each material group (n = 10).

### 2.2. Specimen Preparation

**Figure 2:**
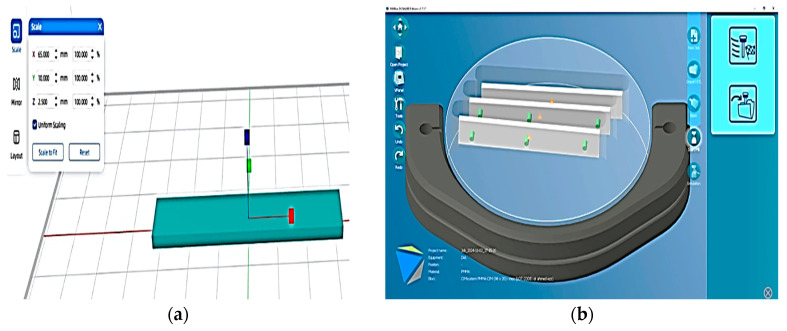
A bar-shaped CAD design for the FS specimen: (**a**) bar-shaped design with dimensions of 65 mm length, 10 mm width, and 2.5 mm thickness; (**b**) STL file sent to CAD/CAM milling machine.

**Figure 3:**
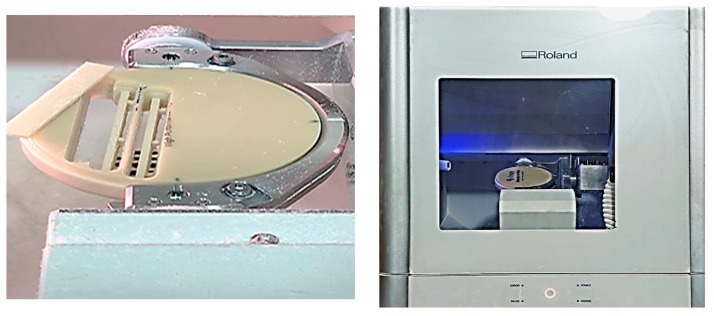
Specimen preparation for FS test using Ronald DWX-51D 5-axis dental milling machine.

CAD software (VPanel for DWX Software-Roland DGA Corporation) was used to design bar shapes with dimensions of 65.0 mm length × 10.0 mm width × 2.5 mm thickness, and the stereolithography (STL) file was then sent to a CAD/CAM milling machine (Figure 2). A 5-axis dental milling machine (Ronald DWX-51D; Ronald DGA corporation, USA) was used to slice each material block into 20 bar-shaped specimens (Figure 3), with dimensions of 65.0 mm length × 10.0 mm width × 2.5 mm thickness (in line with American Dental Association (ADA) Specification No. 12), for the flexural strength test [12,29].

Before flexural strength testing, specimens must be polished and finished in accordance with ISO requirements. Silicon carbide paper with grits of 600, 800, and 1200 was used to finish and polish the specimens while they were continuously water-irrigated with a polishing machine [7]. As the same milling machine was used to prepare the specimens for both materials (Bio-HPP/PEEK and Nanoksa G-plus) in this investigation, machine tolerance was disregarded in order to prevent bias in machine precision. The final measurements were verified using a digital calliper (Adoric Electronic Digital Caliper, Adoriclife Corporation, Orlando, FL, USA) at three separate locations, 10 mm from each specimen’s ends and in the centre. The confirmation of unvarying specimen dimensions eliminated any variability that may have impacted the results by guaranteeing that the specimens had the same cross-sectional area and dimensions. After that, the specimens were examined visually for cracks, flaws, and fractures. Defective specimens were discarded (there were none in the current investigation) [7].

### 2.3. Thermal Ageing Procedure

**Figure 4:**
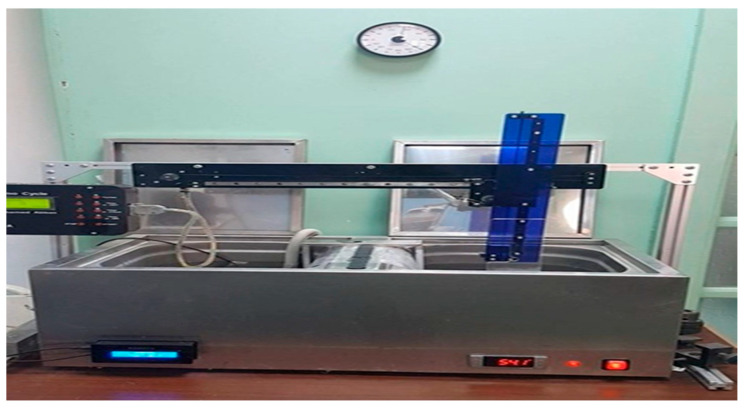
Flexural strength test (3-point bending test) using Instron universal testing machine.

To examine the effects of thermal cycling, ten specimens made of Nanoksa G-plus and Bio-HPP/PEEK (for flexural and hardness) were thermocycled 5000 times (corresponding to six months of clinical use) in distilled water between 5 °C and 55 °C using a thermocycler device (Robota automated thermal cycler; BILGE, Turkey), as shown in Figure 4. Each temperature included a 15 s rest period, and switching between the hot and cold-water baths took 15 s. After thermocycling, ten specimens of each material (Nanoksa G-plus and Bio-HPP/PEEK) were tested for flexural strength, and ten more were tested for hardness. Following thermocycling, the TA specimens were stored for 24 h in distilled water at 37 °C before being analysed [7,29,30].

### 2.4. Flexural Strength Testing Procedures

**Figure 5:**
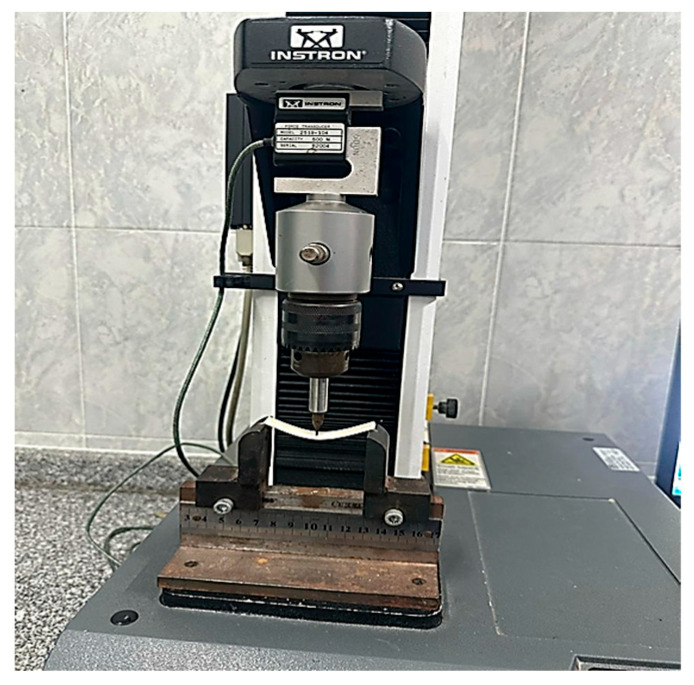
Flexural strength test (3 pint bending test) by Instron universal testing machine.

After being prepared, the NTA specimens were stored for 24 h in distilled water at 37 °C before being tested. Using an Instron universal testing machine (3345 Series, Instron, Norwood, MA, USA), each specimen was subjected to a 3-point bending test (Figure 5), with a crosshead speed of 0.5 mm/min in a 500 newton (N) cell. Static stress was applied to each specimen in the Instron universal testing machine until it broke completely [5,29].

Flexural strength was calculated in megapascal (MPa) using the values obtained from the 3-point bending test and the following equation [5,7,12,26]:

σ = 3Fl/2wh2,(1)

Here, σ is the flexural strength, F is the maximum applied load (N), l is the span length (60.0 mm) between the supports, w is the specimen’s width (10.0 mm), and h is the specimen’s height (2.5 mm).

### 2.5. Vickers Microhardness Testing Procedures

**Figure 6:**
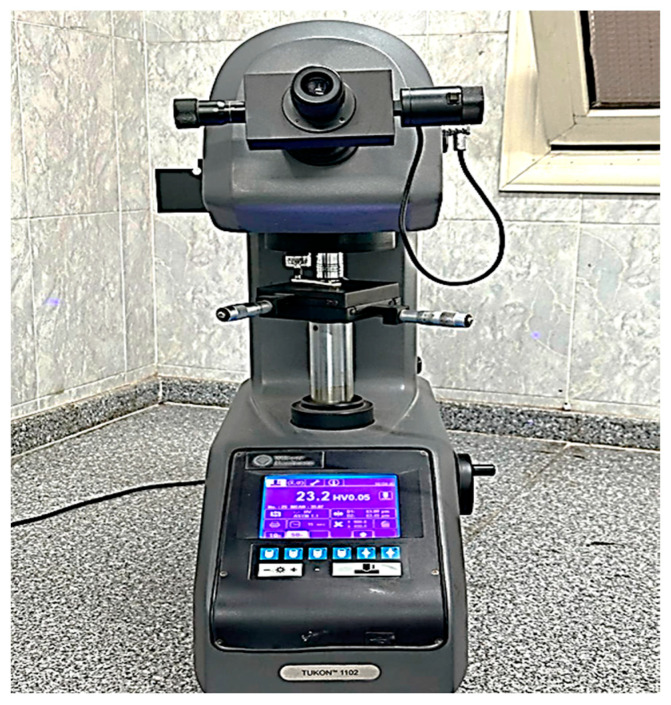
Vickers hardness test using Tukon 1102 Vickers hardness tester.

Every specimen that broke during the flexural strength test was subjected to microhardness measurement. A Vickers hardness tester with a digital camera was used to measure the hardness at five different sites on each specimen (thus recording the Vickers hardness five times per specimen), which were 2 mm apart and 5 mm apart from the line of fracture. Using a 60 gf load and a 15 s dwell duration, the microhardness measurements were carried out using the Vickers hardness tester (Tukon 1102, Instron^®^/TW Company, USA) (Figure 6). The Vickers hardness (HV) value was determined using the following equation [2,12,27]:

HV = 1.8544(F/d2),(2)

Here, d is the diagonal length of the indentation, F is the load, and HV is the Vickers hardness number. The mean of the 5 microhardness readings was considered the hardness value of the specimen.

### 2.6. Statistical Analysis

An IBM-compatible personal computer running SPSS Statistical Package of Social Science version 20 (SPSS Inc. Chicago, Released 201, Version 20.0 of IBM SPSS statistics for Windows, Armonk, NY, USA: IBM Corp.) was used to statistically analyse the collected results. The normality assumption was checked using the Shapiro–Wilk test. Before and after thermal ageing, the two types of high-performance polymers were compared for statistical significance using the *t*-test.

## 3. Results

### 3.1. Flexural Strength Results

The independent *t*-test results showed a statistically significant difference (*p* < 0.0001) between the mean flexural strength values of Nanoksa G-Plus and Bio-HPP/PEEK before thermal ageing. Bio-HPP/PEEK exhibited a significantly higher mean flexural strength value than Nanoksa G-Plus before thermal ageing (Table 1).

Moreover, the independent *t*-test results showed a statistically significant difference (*p* < 0.0001) between the mean flexural strength values of Nanoksa G-Plus and Bio-HPP/PEEK after thermal ageing. The mean flexural strength value of Bio-HPP/PEEK was significantly greater than that of Nanoksa G-Plus following thermal ageing (Table 1).

### 3.2. Microhardness Results

The independent *t*-test results showed a statistically significant difference (*p* < 0.0001) between the mean Vickers microhardness values of Nanoksa G-Plus and Bio-HPP/PEEK before thermal ageing. Before thermal ageing, Bio-HPP/PEEK exhibited a significantly higher mean Vickers microhardness value than Nanoksa G-Plus (Table 2).

Furthermore, the independent *t*-test results showed a significant difference (*p* < 0.0001) between the mean Vickers microhardness values of Nanoksa G-Plus and Bio-HPP/PEEK after thermal ageing. After thermal ageing, Bio-HPP/PEEK showed a significantly higher mean Vickers microhardness value than Nanoksa G-Plus (Table 2).

### 3.3. Effect of Thermal Ageing

The dependent *t*-test results showed a statistically significant difference (*p* < 0.001) between the mean flexural strength values of the Nanoksa G-Plus material before thermal ageing (NTA) and after thermal ageing (TA). The results showed that the mean flexural strength values of the Nanoksa G-Plus material significantly decreased after thermal ageing compared to those before thermal ageing (Table 3).

Moreover, the dependent *t*-test results showed a statistically significant difference (*p* < 0.001) between the mean flexural strength values of the Bio-HPP/PEEK material before thermal ageing (NTA) and after thermal ageing (TA). The results showed that the mean flexural strength values of the Bio-HPP/PEEK material significantly de-creased after thermal ageing compared to those before thermal ageing (Table 3).

The dependent *t*-test results showed that the mean Vickers microhardness values of the Nanoksa G-Plus material before thermal ageing (NTA) and after thermal ageing (TA) differed significantly (*p* = 0.007). The results showed that the mean Vickers microhardness values of the Nanoksa G-Plus material significantly decreased after thermal ageing compared to those before thermal ageing (Table 4).

Furthermore, the dependent *t*-test results showed that the mean Vickers microhardness values of the Bio-HPP/PEEK material before thermal ageing (NTA) and after thermal ageing (TA) differed significantly (*p* = 0.019). The results showed that the mean Vickers microhardness values of the Bio-HPP/PEEK material significantly decreased after thermal ageing compared to those before thermal ageing (Table 4).

## 4. Discussion

The ideal production conditions for the polymeric materials used in CAD/CAM technology provide excellent quality control and conversion rates. As a result, materials with superior mechanical strength, biocompatibility, and structural characteristics are produced [5].

In this study, two CAD/CAM high-performance polymeric materials (Bio-HPP/PEEK and Nanoksa G-Plus) were evaluated in terms of flexural strength and microhardness both before and after thermal ageing. Additionally, the effects of thermal ageing on each polymeric material were examined.

The results of the current study demonstrate that, both before and after thermal ageing, Bio-HPP/PEEK had substantially superior microhardness and flexural strength characteristics compared to Nanoksa G-Plus; thus, the first null hypothesis was rejected.

Moreover, the findings of this study show that the flexural strength and microhardness values of the Bio-HPP/PEEK and Nanoksa G-Plus materials significantly decreased after thermal ageing; as a result, the second null hypothesis was also rejected.

The findings of the current study show that, before thermal ageing, Bio-HPP/PEEK exhibited notably better microhardness and flexural strength characteristics than Nanoksa G-Plus. This may be explained by the fact that the Bio-HPP/PEEK matrix contained an aryl ring with ketone along with ether groups, as well as the addition of 20% zirconia nanoparticle reinforcement, as the filler’s extremely high strength and the matrix’s flawless interfacial bond enhance its mechanical properties [2,11].

Additionally, it has been reported that some nano-powders (such as zirconia) can increase resistance to crack formation, and they also tend to inhibit the growth of microcracks; it is known that the incidence of microcracks in the composite material structure results in a further reduction in the mechanical properties [31].

In unfilled polymers, molecular mobility can occur in more convenient locations. Conversely, in filled polymers, reinforcing components serve as nucleating agents for the production of transcrystalline layers. Thus, molecular mobility is reduced by rein-forcing agents [32]. This could explain the decrease in the microhardness and flexural strength characteristics of Nanoksa G-Plus, as it contains more than one type of rein-forcing agent (nano-zirconia and nano-carbon), which could have decreased the molecular mobility of the polymer chain and produced transcrystalline layers that negatively impacted its physical properties.

However, insufficient information was provided by the manufacturer of Nanoksa G-Plus regarding the filler content and polymer composition. It has been reported that the use of carbon nanotubes in combination with partially stabilised zirconia can inhibit the transformation toughening of zirconia [33]. This could also explain the de-crease in the microhardness and flexural strength characteristics of Nanoksa G-Plus.

Moreover, this might also be explained by variations in the polymer chemistry, degree of cross-linking or crystallinity, existence of an additional inorganic phase, and resistance to fluid sorption of the studied resins. The microstructure of the Bio-HPP/PEEK material employed in this investigation exhibits strong cross-linking with 30–35% crystallinity [3,12].

Given that the degree of crystallinity significantly influences the mechanical characteristics, a material will be harder if its crystalline loading rate is higher [12]. In PAEK, the ether and ketone linkages shown cause chain packing, which causes PAEK polymers to crystallise. Ketone connections are less flexible than ether linkages and slow down crystallisation and chain packing. In comparison to PEKK, PEEK has a lower ketone content and a higher crystallinity, which make it harder and more flexible [7]. This explains why the hardness values of Bio-HPP/PEEK were notably greater than those of Nanoksa G-plus.

Although it is often acknowledged that nanofillers improve the mechanical characteristics of composites, their exceptional qualities remain unrealised. It is anticipated that relative gains in modulus would outweigh increases in tensile strength. In general, strain at break reduces as nanofiller loading increases [34]. The two materials tested in the present investigation may have differed in their surface properties, flexural strength, and reaction to thermal ageing due to their varied matrices, filler types, arrangements, and/or amounts [35]. The Bio-HPP/PEEK material employed in this study had 20% ceramic filler reinforcement with a grain size of 0.3 to 0.5 micrometres [2,3]. Smaller-sized ceramic fillers can enter and seal the gap between the Bio-HPP/PEEK polymer’s chains, limiting chain mobility and lowering the penetration of various ageing treatments [3]. However, although the Nanoksa G-Plus polymer is reinforced with nano-zirconia and nano-carbon, details of the exact percentage and agglomeration are unknown.

Thermocycling is one of the most popular techniques for ageing resin-based mate-rials because it produces temperature changes in an experimental setting that are quite close to those in actual oral settings. During the thermocycling process, test specimens are submerged in hot and cold distilled water to replicate temperature cycles for pre-determined lengths of time [2,25,36]. The temperature range for thermal cycling is 5–55 °C. This is defined as the range of temperatures for different dietary items, such as hot soup, cold drinks, and beverages [3].

The results of this investigation revealed that the flexural strength and microhardness values of Bio-HPP/PEEK and Nanoksa G-Plus materials significantly decreased after thermal ageing. According to previous reports, resinous composite materials (such as Bio-HPP/PEEK and Nanoksa G-Plus materials in the present study) showed signs of deterioration when subjected to thermocycling [2,36]. This phenome-non could be attributed to variations in the resinous composite materials’ matrices and the fillers’ thermal expansion and contraction coefficients. The resinous composites developed internal strains as a result of the temperature fluctuations during thermo-cycling, which led to the formation of tiny microcracks. Additionally, they ab-sorbed water due to their hydrophilic nature and the formation of these tiny microcracks. Therefore, they degraded as a result of all these variables [2,25,36,37].

However, the reinforcement of PEEK improves its mechanical characteristics [2]. The ceramic filler reinforcement in Bio-HPP/PEEK and Nanoksa G-Plus materials produces a complex system, and it adversely affected their microhardness and flexural strength characteristics after thermal ageing in the present investigation [2,26,38].

In accordance with the findings of the present study, previous investigations found that, during the ageing process, the stability of the mechanical characteristics of reinforced PEEK was impacted more than that of unfilled PEEK [2,26]. The results of this study are also consistent with those of a previous study that found that thermocycling had a negative impact on the marginal integrity of crowns made with milled hybrid resin–ceramic materials (Nanoksa G-plus, IONX) and 3D printed hybrid resin–ceramic materials (Nanoksa Bioguard, INOX) after 5000 cycles [37]. This indicates that the Nanoksa G-plus polymer is negatively impacted by thermocycling.

Previous research has shown that the thermal stress created between composite components can result in microcracks and that the temperature gradient and water absorbed by dental composites during the thermocycling process alter their material properties, causing the degradation of their surfaces [2,25,36].

Furthermore, the existence of any damaged bonding sites or flaws exacerbates fluid absorption at the interfacial zones. Through hydrolysis and osmotic cracking, the absorbed fluid particles may cause the resin phase to plasticise and the macromolecular skeleton to become embrittled [3,38].

This study shed important light on the longevity of high-performance polymeric materials that are biocompatible and metal-free and may be used to make denture frameworks. This study also investigated the reactions of the materials in ageing simulations. Prosthodontic practitioners can use this knowledge to guide the selection of appropriate materials for use in various clinical situations.

Different multidirectional pressures are applied to CAD/CAM-milled dental restorations during intraoral function. Even though the test specimens were prepared and finished according to a defined technique, internal porosity could not be controlled because of potential manufacturing mistakes in the material blocks, production faults, and stress release during the polishing and finishing processes [5].

The limitations of the study include the fact that only a few characteristics (microhardness and flexural strength) and a limited number of materials (Bio-HPP/PEEK and Nanoksa G-Plus) were examined. In the current study, only two mechanical properties were assessed under controlled conditions; other parameters, including dimensional stability, colour, roughness, and sensitivity to cyclic loading, are equally important in determining an HPP’s clinical performance.

Future research is recommended to take into account the long-term impact and interactions of additional variables, including surface roughness, colour stability, and cyclic stress, on how dental high-performance polymer materials age in relation to the complex oral environment.

## 5. Conclusions

Based on the investigated parameters and limitations of the current study, the following conclusions are drawn:

1. Compared to Nanoksa G-Plus, Bio-HPP/PEEK demonstrated superior microhardness and flexural strength characteristics before and after thermal ageing.
2. The flexural strength and microhardness of Bio-HPP/PEEK and Nanoksa G-Plus were significantly reduced by thermal ageing.
3. Bio-HPP/PEEK might be a good substitute for Nanoksa G-Plus, particularly for long-term denture frameworks and applications that require high wear resistance, such as telescopic attachments. However, such compelling claims need to be supported by more thorough clinical and mechanical evaluations.
4. Future applications of Bio-HPP/PEEK and Nanoksa G-Plus materials in numerous clinical dental contexts are made possible by their acceptable mechanical characteristics and other clinical, aesthetic, and financial considerations.

## Figures and Tables

**Table 1:** Flexural strength results (MPa) of Nanoksa G-Plus and Bio-HPP/PEEK before and after thermal ageing.

Flexural Strength	Nanoksa G-Plus (Mean ± SD)	Bio-HPP/PEEK (Mean ± SD)	MD	T-Value	*p*-Value
NTA	97.72 ± 1.27	126.78 ± 2.87	−29.06	−29.28	<0.0001 *
TA	78.71 ± 1.18	111.90 ± 1.83	−33.2	−48.20	<0.0001 *

* Significant at *p* < 0.05. SD, standard deviation. TA, thermal ageing. NTA, no thermal ageing. MD, mean difference.

**Table 2:** Microhardness results (VHN) of Nanoksa G-Plus and Bio-HPP/PEEK before and after thermal ageing.

Microhardness	Nanoksa G-Plus (Mean ± SD)	Bio-HPP/PEEK (Mean ± SD)	MD	T-Value	*p*-Value
NTA	17.59 ± 0.61	23.33 ± 0.58	−5.74	−21.522	<0.0001 *
TA	12.61 ± 0.75	19.43 ± 0.64	−6.82	−21.63	<0.0001 *

* Significant at *p* < 0.05. SD, standard deviation. TA, thermal ageing. NTA, no thermal ageing. MD, mean difference.

**Table 3:** Effect of thermal ageing on the flexural strength (MPa) of Nanoksa G-Plus and Bio-HPP/PEEK materials.

Flexural Strength	NTA (Mean ± SD)	TA (Mean ± SD)	MD	T-Value	*p*-Value
Nanoksa G-Plus	97.72 ± 1.27	78.71 ± 1.18	−13.83	−5.63	<0.001 *
Bio-HPP/PEEK	126.78 ± 2.87	111.90± 1.83	−11.62	−5.33	<0.001 *

* Significant at *p* < 0.05. SD, standard deviation. TA, thermal ageing. NTA, no thermal ageing. MD, mean difference.

**Table 4:** Effect of thermal ageing on the Vickers microhardness (VHN) of Nanoksa G-Plus and Bio-HPP/PEEK materials.

Microhardness	NTA (Mean ± SD)	TA (Mean ± SD)	MD	T-Value	*p*-Value
Nanoksa G-Plus	17.59 ± 0.61	12.61 ± 0.75	−4.93	−3.20	0.007 *
Bio-HPP/PEEK	23.33 ± 0.58	19.43 ± 0.64	−4.109	−2.67	0.019 *

* Significant at *p* < 0.05. SD, standard deviation. TA, thermal ageing. NTA, no thermal ageing. MD, mean difference.

## Data Availability

The raw data supporting the conclusions of this article will be made available by the authors on request.

## Abbreviations

The following abbreviations are used in this manuscript:

CAD/CAM	Computer-Aided Design/Computer-Aided Manufacturing
FS	Flexural Strength
HPP	High-Performance Polymer
HV	Vickers hardness
ISO	International Organization for Standardization
MD	Mean Difference
NTA	No Thermal Ageing
PAEK	Poly-Aryl-Ether-Ketone
PEEK	Poly-Ether-Ether-Ketone
STL	Stereolithography
SD	Standard Deviation
TA	Thermal Ageing

## References

[1] Priester M., Müller W.D., Beuer F., Schmidt F., Schwitalla A.D. (2021). Performance of PEEK based telescopic crowns; a comparative study. Dent. Mater..

[2] Porojan L., Toma F.R., Bîrdeanu M.I., Vasiliu R.D., Uțu I.D., Matichescu A. (2022). Surface characteristics and color stability of dental PEEK related to water saturation and thermal cycling. Polymers.

[3] Fathy S., Emera R., Abdallah R. (2021). Surface microhardness; flexural strength; and clasp retention and deformation of acetal vs. poly-ether-ether ketone after combined thermal cycling and pH aging. J. Contemp. Dent. Pract..

[4] Skirbutis G., Dzingutė A., Masiliūnaitė V., Šulcaitė G., Žilinskas J. (2017). A review of PEEK polymer’s properties and its use in prosthodontics. Stomatologija.

[5] Yeslam H. (2023). Flexural behavior of biocompatible high-performance polymer composites for CAD/CAM dentistry. J. Compos. Sci..

[6] Porto T.S., Roperto R.C., Akkus A., Akkus O., Teich S., Faddoul F., Porto-Neto S.T., Campos E.A. (2019). Effect of storage and aging conditions on the flexural strength and flexural modulus of CAD/CAM materials. Dent. Mater. J..

[7] Yeslam H.E., Alharbi S., Albalawi W., Hasanain F.A. (2023). The effect of thermal aging on flexural strength of CAD/CAM hybrid and polymeric materials. Mater. Res. Express.

[8] Manziuc M., Khechen A.A., Negucioiu M., Poiană I., Kui A., Mesaroș A., Buduru S. (2023). Survival rates of glass versus hybrid ceramics in partial prosthetic restorations: A scoping review with emphasis on adhesive protocols. J. Clin. Med..

[9] Singh R.P., Zhang M., Chan D. (2002). Toughening of a brittle thermosetting polymer, Effects of reinforcement particle size and volume fraction. J. Mater. Sci..

[10] Lo Giudice R., Sindoni A., Tribst J.P.M., Dal Piva A.M.d.O., Lo Giudice G., Bellezza U., Lo Giudice G., Famà F. (2022). Evaluation of zirconia and high performance polymer abutment surface roughness and stress concentration for implant-supported fixed dental prostheses. Coatings.

[11] Alam M.A., Samad U.A., Anis A., Sherif E.M., Abdo H.S., Al-Zahrani S.M. (2023). The effect of zirconia nanoparticles on thermal; mechanical; and corrosion behavior of nanocomposite epoxy coatings on steel substrates. Materials.

[12] Emera R.M.K., Abdallah R.M. (2021). Denture base adaptation; retention; and mechanical properties of BioHPP versus nano-alumina-modified polyamide resins. J. Dent. Res. Dent. Clin. Dent. Prospects.

[13] Zol S.M., Alauddin M.S., Said Z., Mohd Ghazali M.I., Hao-Ern L., Mohd Farid D.A., Zahari N.A.H., Al-Khadim A.H.A., Abdul Aziz A.H. (2023). Description of poly(aryl-ether-ketone) materials (PAEKs), polyetheretherketone (PEEK) and polyetherketoneketone (PEKK) for application as a dental material: A materials science review. Polymers.

[14] GROUPS NGPUSD Nanoksa-g-plus, i-noxmena.net. https://i-noxmena.net.

[15] Nanoksa BioGuard Resin Advanced Resin for 3D Printing Dentistry. https://shop.v-ceram.com/shop/nanoksa-bioguard-resin/.

[16] SIST EN ISO 527-1:2019—Plastics—Determination of Tensile Properties—Part 1: General Principles (ISO 527-1:2019)—USA. https://www.iso.org/standard/527-1.

[17] SIST EN ISO 178:2019—Plastics—Determination of Flexural Properties—Part 1: General Principles (ISO 178:2019)—USA. https://www.iso.org/obp/ui/#iso:std:iso:178:ed-6:v1:en.

[18] Precision and Longevity in Every Nanoksa G Plus Disc. https://i-nox.us/nanoksa-g-plus-disc/.

[19] Choi Y.-S., Kang K.-H., Att W. (2020). Effect of aging process on some properties of conventional and multilayered translucent zirconia for monolithic restorations. Ceram. Int..

[20] Li Q., Zhao W., Li Y., Yang W., Wang G. (2019). Flexural properties and fracture behavior of CF/PEEK in orthogonal building orientation by FDM, microstructure and mechanism. Polymers.

[21] Broitman E. (2016). Indentation hardness measurements at macro-, micro-, and nanoscale: A critical overview. Tribol. Lett..

[22] Wassell R.W., McCabem J.F., Walls A.W. (1992). Subsurface deformation associated with hardness measurements of composites. Dent. Mater..

[23] Dionysopoulos D., Gerasimidou O. (2021). Wear of contemporary dental composite resin restorations, a literature review. Restor. Dent. Endod..

[24] Willems G., Celis J.P., Lambrechts P., Braem M., Vanherle G. (1993). Hardness and Young’s modulus determined by nanoindentation technique of filler particles of dental restorative materials compared with human enamel. J. Biomed. Mater. Res..

[25] Kim S.Y., Bae H.J., Lee H.H., Lee J.H., Kim Y.J., Choi Y.S., Lee J.H., Shin S.Y. (2023). The effects of thermocycling on the physical properties and biocompatibilities of various CAD/CAM restorative materials. Pharmaceutics.

[26] Gao S., Gao S., Xu B., Yu H. (2015). Effects of different pH-values on the nanomechanical surface properties of PEEK and CFR-PEEK compared to dental resin-based materials. Materials.

[27] Sonmez N., Gultekin P., Turp V., Akgungor G., Sen D., Mijiritsky E. (2018). Evaluation of five CAD/CAM materials by microstructural characterization and mechanical tests, a comparative in vitro study. BMC Oral Health.

[28] Faul F., Erdfelder E., Buchner A., Lang A.G. (2009). Statistical power analyses using G*Power 3.1, tests for correlation and regression analyses. Behav. Res. Methods.

[29] Kim S.-H., Choi Y.-S., Kang K.-H., Att W. (2022). Effects of thermal and mechanical cycling on the mechanical strength and surface properties of dental CAD-CAM restorative materials. J. Prosthet. Dent..

[30] Egilmez F., Ergun G., Cekic-Nagas I., Vallittu P.K., Lassila L.V.J. (2018). Does artificial aging affect mechanical properties of CAD/CAM composite materials. J. Prosthodont. Res..

[31] Salih S.I., Braihi A.J., Sadeq H.M. (2020). Comparative study of some mechanical properties of nanocomposites based on the polymer’s blends used for dentures base applications. Mater. Res. Express.

[32] Sınmazçelik T., Yılmaz T. (2007). Thermal aging effects on mechanical and tribological performance of PEEK and short fiber reinforced PEEK composites. Mater. Des..

[33] Mohapatra P., Rawat S., Mahato N., Balani K. (2015). Restriction of phase transformation in carbon nanotube-reinforced yttria-stabilized zirconia. Metallurg. Mater. Trans. A.

[34] Bhattacharya M. (2016). Polymer nanocomposites—A comparison between carbon nanotubes; graphene; and clay as nanofillers. Materials.

[35] Babaier R.S., Haider J., Alshabib A., Silikas N., Watts D.C. (2022). Mechanical behaviour of prosthodontic CAD/CAM polymer composites aged in three food-simulating liquids. Dent. Mater..

[36] Yan Y., Chen C., Chen B., Shen J., Zhang H., Xie H. (2020). Effects of hydrothermal aging; thermal cycling; and water storage on the mechanical properties of a machinable resin-based composite containing nano-zirconia fillers. J. Mech. Behav. Biomed. Mater..

[37] Sayed A.M., Odeh E. (2025). The effect of thermocycling on the marginal integrity of 3d printed and machined hybrid resin-ceramic crowns: An in-vitro study. Egypt. Dent. J..

[38] Sethi S., Ray B.C. (2015). Environmental effects on fibre reinforced polymeric composites, evolving reasons and remarks on interfacial strength and stability. Adv. Colloid Interface Sci..

